# Preschool children’s temperament and its associations with energy intake: Riikka Pajulahti

**DOI:** 10.1093/eurpub/ckac129.693

**Published:** 2022-10-25

**Authors:** R Pajulahti, C Ray, K Salmela-Aro, L Korkalo, R Lehto, H Vepsäläinen, K Nissinen, E Roos, M Erkkola

**Affiliations:** 1 Department of Food and Nutrition, University of Helsinki, Helsinki, Finland; 2 Folkhälsan Research Center, Helsinki, Finland; 3 Faculty of Educational Sciences, University of Helsinki, Helsinki, Finland; 4 Seinäjoki University of Applied Sciences, Helsinki, Finland; 5 Department of Public Health, University of Helsinki, Helsinki, Finland

## Abstract

**Background and objectives:**

Child's temperament dimensions have been linked with different weight outcomes as well as dietary factors such as consumption of fruit and vegetables, sugar-rich foods and drinks and an overall dietary quality. Links between temperament dimensions and energy intake, however, remain under-examined. This study expands the literature by investigating associations between child's temperament dimensions and energy intake.

**Methods:**

Altogether 505 Finnish children aged 3-6 years provided data for the analyses. The data is a part of the DAGIS (Increased Health and Wellbeing in Preschools) study conducted in 2015-2016. Child's energy intake was measured with 3-day food records. To be included, children had to 1) have food record for two preschool days and one weekend day and 2) to have consumed both lunch and an afternoon snack in the preschool on the two preschool days. The very short form of Children's Behavior Questionnaire was used to measure child's temperament dimensions. Concurrent associations between three temperament dimensions (surgency, negative affectivity, and effortful control) and energy intake were examined using linear regression models adjusted for child's age, sex, mother's highest education, and moderate-to-vigorous physical activity.

**Results:**

Surgency, temperament dimension referring to characteristics such as impulsivity, high activity level and high approach, was positively associated with energy intake. Effortful control or negative affectivity were not associated with energy intake.

**Conclusions:**

The findings imply that temperamental surgency may be one relevant determinant of energy intake among preschool children. The result is in line with previous studies linking child's surgency as well as its specific facets, such as impulsivity, with weight outcomes and food approach behaviors. Considering child's individual temperament dimensions when counselling families in eating issues could be beneficial.

**Key messages:**

• Surgency, a temperament dimension referring to characteristics such as impulsivity, high activity level and high approach, was positively associated with energy intake.

• When promoting children’s balanced eating, tailored support for parents acknowledging child’s temperament could be beneficial.

